# The Effects of the Biological Agents Infliximab, Vedolizumab, and Ustekinumab on Intestinal Anastomosis: An Experimental Study in Rats

**DOI:** 10.3390/biomedicines13051079

**Published:** 2025-04-29

**Authors:** Alexandra Menni, Georgios Tzikos, Patroklos Goulas, George Chatziantoniou, Angeliki Vouchara, Athanasios S. Apostolidis, Aristeidis Ioannidis, Georgios Germanidis, Lyssimachos G. Papazoglou, Olga Giouleme, Stylianos Apostolidis

**Affiliations:** 1 1st Propaedeutic Department of Surgery, AHEPA University Hospital of Thessaloniki, 546 36 Thessaloniki, Greece; tzikos_giorgos@outlook.com (G.T.); patroklos@live.com (P.G.); georchantziantoniou@gmail.com (G.C.); aggvou9@gmail.com (A.V.); thanasisapostolidis@outlook.com (A.S.A.); ariioann@yahoo.gr (A.I.); stlsa@auth.gr (S.A.); 2 Laboratory of General Pathology & Pathological Anatomy, Department of Pathology, Aristotle University of Thessaloniki, 541 24 Thessaloniki, Greece; 3 1st Department of Internal Medicine, AHEPA University Hospital of Thessaloniki, 546 36 Thessaloniki, Greece; geogerm@auth.gr; 4 School of Veterinary Medicine, Faculty of Health Sciences, Aristotle University of Thessaloniki, 541 24 Thessaloniki, Greece; makdvm@vet.auth.gr; 5 2nd Propaedeutic Department of Internal Medicine, Hippokrateion University Hospital of Thessaloniki, 546 42 Thessaloniki, Greece; giouleme@auth.gr

**Keywords:** IBDs, biological agents, infliximab, vedolizumab, ustekinumab, experimental study, surgical complications

## Abstract

**Background/Objectives**: The potential side effects of the use of biological agents in the perioperative period are still under investigation. This animal prospective study aimed to evaluate the overall impact of biological factor administration after intestinal surgery. **Methods**: This study included 80 female Wistar rats sorted into four groups: three groups received one of the biological factors, infliximab, vedolizumab, or ustekinumab; the control group received placebo therapy. After enterotomy and intestinal anastomosis, the bursting pressure (BP) of the anastomosis was compared among the groups on postoperative days (PODs) 3 and 7. **Results**: On POD3, the control group presented with a significantly higher mean BP (154.6 ± 39.7 mmHg) compared to the infliximab (66.8 ± 10.4 mmHg), vedolizumab (105.4 ± 37.6 mmHg), and ustekinumab (98.8 ± 47.9 mmHg) groups. A post hoc analysis among the three biological agent groups revealed differences only when comparing infliximab and vedolizumab rats with the controls on POD3 (*p* < 0.001) and with the ustekinumab rats on POD7, having a greater mean BP (282.5 ± 80.1 mmHg, *p* = 0.031). No differences were observed regarding the event of broken anastomosis among the four groups. **Conclusions**: This experimental study’s findings highlight the varying detrimental effects of different biological agents on the strength of intestinal anastomosis, with ustekinumab demonstrating superior performance.

## 1. Introduction

Inflammatory bowel diseases (IBDs) are autoimmune conditions, and their rate of diagnosis has been increasing over the years. In the pre-biologic era, up to 80% of patients required surgery to manage the disease and its complications [1]. Since the introduction of anti-TNF agents, the need for surgical intervention has significantly decreased [2]. Specifically, the early initiation of anti-TNFs in combination with an immunomodulator reduces the rates of surgery, hospitalization, and serious disease-related complications from 35.1% to 27.7% over a 2-year period after initiation [3]. However, one-third of patients with IBDs do not respond to anti-TNF agents due to non-TNF-mediated inflammatory pathways, and even one-third of initially responsive patients lose the ability to respond to therapy due to reduced drug levels or the development of antibodies against the agents [4].

Until 2014, non-anti-TNF therapies for IBDs were limited, but the approval of vedolizumab (VDLZ), an α4β7 integrin inhibitor, marked a significant development. Subsequently, in 2016, ustekinumab (USTK), a monoclonal antibody targeting interleukins 12 and 23, was approved for the treatment of moderate to severe Crohn’s disease; USTK had already been available since 2009 for patients with psoriasis [5]. As a result, approximately 30–50% of patients are likely to be receiving a biologic agent at the time of surgery [6].

Data on the risk of perioperative infections and postoperative complications associated with the use of anti-TNF agents are conflicting, largely due to the considerable heterogeneity of individual studies [7]. A meta-analysis of eight studies reported a trend toward an increase in overall complications in patients with the preoperative use of anti-TNF agents [8]. Specifically, regarding the administration of infliximab, no statistically significant differences were found between the two groups in terms of the peak breakdown pressure, inflammation parameters, and collagen formation in the anastomosis region [9,10]. In comparison, the preoperative use of VDLZ is relatively safer in adult patients with IBDs, as it does not increase the risk of overall postoperative complications. However, it does increase the risk of all surgical site infections (SSIs) and the incidence of postoperative ileus [11]. Finally, the rate of postoperative complications in patients with preoperative USTK exposure may be similar to that with anti-TNF agents [12]. In contrast, a univariate analysis showed that subjects treated with vedolizumab had a higher rate of postoperative complications and ileus compared to patients preoperatively administered ustekinumab [13]. Therefore, while preoperative exposure to USTK may influence the postoperative risk of complications to some extent, the results remain inconclusive, and further studies are necessary. Consequently, the evidence remains conflicting.

Various studies have investigated the effects of biological agents, monoclonal antibodies, and anti-TNFs on surgical anastomoses. Most of these studies focused on anti-TNF agents (including infliximab), with fewer investigating VDLZ, and even fewer exploring USTK. Furthermore, the studies generally focused on overall postoperative complications and did not necessarily address intestinal healing. Very few studies have simultaneously compared all three categories of biological therapies. One experimental study investigated the effect of infliximab administration in rats [14], and another examined adalimumab in rabbits [15]. Due to the conflicting and often inconclusive findings in the international literature regarding the impact of biologic agents on postoperative complications, the objective of this study was to provide clarification on these effects. Establishing definitive conclusions would aid clinical practice by offering evidence-based guidelines for the perioperative management of these patients, ultimately improving their postoperative outcomes and reducing their morbidity.

This protocol represents the first experimental study comparing three agents—infliximab, vedolizumab, and ustekinumab—in a laboratory setting, with the primary outcome being the bursting pressure of intestinal anastomoses. These three factors are the treatment of choice in many cases of patients with IBDs, both Crohn’s disease and ulcerative colitis, because they are widely available in Greece at the time of our experiment. Our experimental animal study aims to clarify and investigate the overall impact of administering biological agents (infliximab, vedolizumab, and ustekinumab) during the perioperative period, specifically targeting intestinal anastomoses, and to compare the outcomes with those reported in the existing literature.

## 2. Materials and Methods

### 2.1. Study Design and Ethics

This was a randomized-design experimental study. The main question of this study was the impact of three biological agents on intestinal anastomoses. The primary outcome parameter for intestinal anastomotic healing is the bursting pressure. The bursting pressure, defined as the internal pressure at which an anastomosis fails, is a crucial measure of the mechanical strength of these junctions [16]. Greater bursting pressure was linked to more robust anastomoses in the first week after surgery. Furthermore, we observed the incidence of other postoperative complications such as skin wound infections, incision disruptions, ileus, or intrabdominal adhesions, and, further, the overall mortality of the animals.

The experimental protocol was approved by the Animal Ethics Committee of the Veterinary School and the Bioethics Committee of the Medical School of the Aristotle University of Thessaloniki (protocol No.: 10/2023, 8 November 2023), Greece. Based on the existing data [12,17,18], the sample size for this study was calculated by testing a hypothesis of a minimum difference among four groups of up to 15% concerning the bursting pressure (BP) measured on postoperative days (PODs) 3 and 7. With this assumption, combined with a level of significance set at 5% (α = 0.05), and in order for a study power of 80% to be assumed, a sample of 80 animals was recruited.

### 2.2. Experimental Protocol

#### 2.2.1. Rat Experimental Model and Administration Protocol

The animals considered suitable for this study were Wistar rats obtained from the Veterinary School of the Aristotle University of Thessaloniki. They were female, weighed about 270 to 380 gr, and lived in threes in Plexiglas cages under controlled conditions of light (12 h light–dark cycle) and temperature (21 °C) with free access to food and water for the overall duration of the experiment. There were no other exclusion criteria or any confounders to be controlled. The allocation of the groups was made randomly by the laboratory supervisor, who placed them in groups of 3 in each cage. The team of researchers carrying out the experiments assigned a characteristic number to each animal that identified the cage number (e.g., 1.2, 3.1, 18.3). This number defined the animal until the end of the experiment.

The animals were divided into 4 equal (20-member) groups. The control group (CNTRL-group) consisted of 20 rats in which 2 mL of N/S 0.9% was administered intraperitoneally. The infliximab group (INFL-group), the vedolizumab group (VDLZ-group), and the ustekinumab group (USTK-group) included rats that received customized doses of these biological agents. All agents were administered intraperitoneally. Regarding the doses given, 1. infliximab was administered at a dose of 5 mg/kg body weight, similar to the human indication dose. Each vial contained 100 mg/10 mL of infliximab. After reconstitution, each milliliter contained 10 mg of infliximab. 2. Concerning vedolizumab, the vial used contained 300 mg in 5 mL of total volume; after reconstitution, the concentration of the drug was 60 mg/1 mL. In human patients, the dose of administration is standardized at 300 mg. Based on studies on the experimental stages of the biological agent, the dosage used was 30 mg/kg body weight. 3. Regarding ustekinumab, no existing data for the proper experimental dosage in animals were found in the international literature. However, according to the dosing instructions for humans, for patients weighing ≤55 kg, the dosage is 260 mg; for those weighing >55 kg and ≤85 kg, the corresponding dose is 390 mg; and finally, for those weighing >85 kg, it is 520 mg. Thus, we decided to administer the lowest dose (260 mg for <55 Kg) expressed in mg per kilo, meaning that a dose of 260 mg/55 kg = 4.7 mg/kg body weight was administered. The available vial contained 130 mg of ustekinumab in 26 mL (5 mg/mL).

The rats of each group underwent surgery. For group B, C, and D rats, surgery was performed after the half-life of each drug was administered, in relevance to its maximum concentration. The median terminal half-life of infliximab is 7.7 to 9.5 days. On the other hand, vedolizumab has a long terminal elimination half-life of 25 days, and finally, the estimated median terminal half-life of ustekinumab is approximately 19 days in patients with Crohn’s disease or ulcerative colitis [19].

#### 2.2.2. Bursting Pressure Measurement

On day 0, the rats were anesthetized via the administration of xylazine and ketamine in solutions ready for injection with concentrations of 20 mg/mL and 100 mg/mL, respectively, in each vial. The drugs were administered intraperitoneally in predetermined dosages according to the animals’ body weight. In particular, xylazine was administered in a concentration of 5 mg/Kg body weight (BD) and ketamine’s dosage was 50 mg/Kg BD. Afterwards, and under anesthesia, a small bowel enterotomy at the terminal ileum was performed. Following this, an end-to-end anastomosis with individual full-thickness sutures, with internal rotation, and using a 6-0 sterile synthetic absorbable monofilament suture made from the polyester poly(p-dioxanone) (PDS) was conducted. Closure of the abdominal wall was performed with continuous stitching with 4-0 PDS sutures, and similarly, skin closure was performed with a continuous 4-0 silk suture.

**Figure 1:**
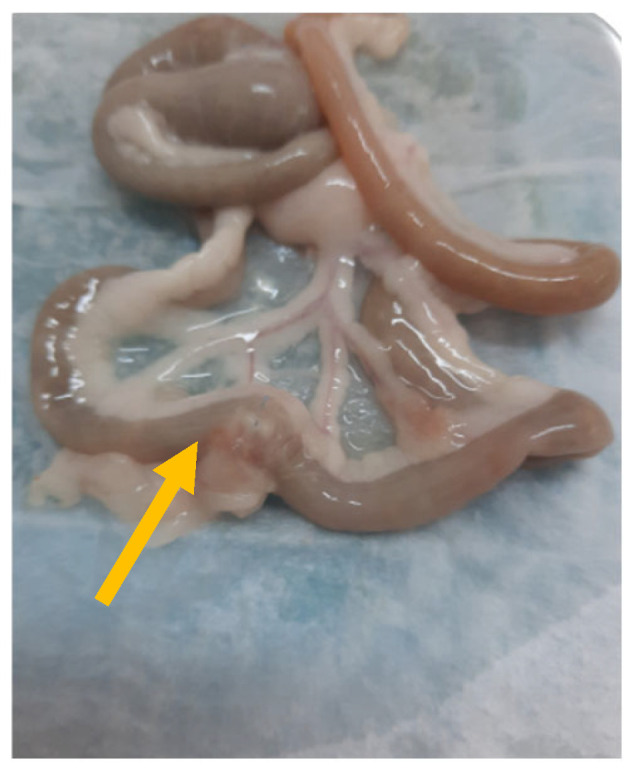
Excised part of intestine containing anastomosis (marked with arrow).

**Figure 2:**
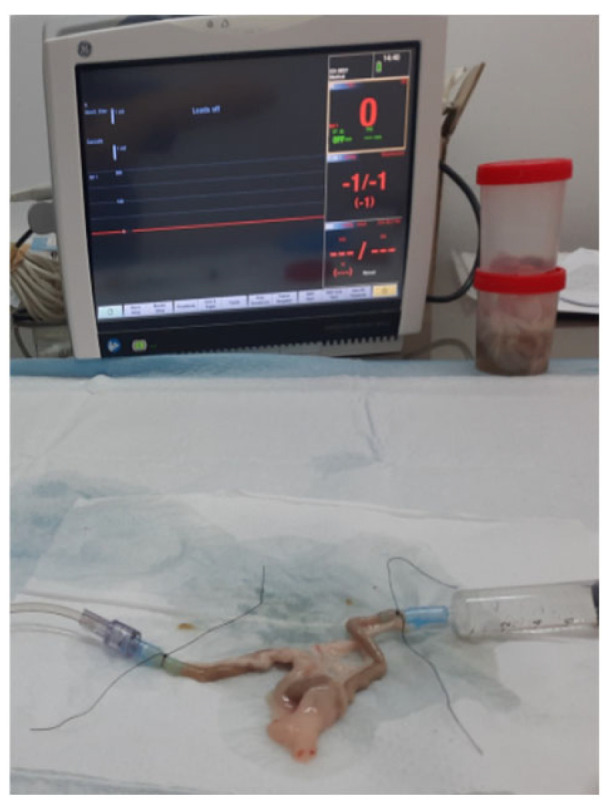
Bursting pressure measurement setting.

On POD3, half of the rats from each group (10 rats from each) were sacrificed, while the remaining half were sacrificed on POD7. After the rats were killed by means of exsanguination, the part of the intestine containing the anastomosis was removed (Figure 1), and blood samples from the inferior vena cava and the right cavities of the heart were collected. In order for the BP to be measured, we proceeded with the construction of the following structure. One 16 G abbocath-T (abboA) was connected to an arterial blood pressure measurement system, and a second 16 G abbocath-T (abboB) was connected to a 60 mL syringe containing normal 0.9% saline solution. Subsequently, at both ends of the part of the intestine including the anastomosis, the two abbocaths-T were placed and secured with No1 silk suture (Figure 2). Next, a slow manual infusion of saline solution was initiated. The saline gradually filled the lumen of the intestine, increasing the intraluminal pressure, which was documented using the blood pressure monitor. Anastomotic rupture was assumed at the point when normal saline escaped the anastomosis, with a simultaneous sharp drop in the pressure curve and a zeroing of the pressure value. The value immediately before this drop constituted the BP.

It is very important to note that intraperitoneal sepsis with the presence of peritonitis or abscesses was not observed in any animal. In some cases, during resection of the intestinal segment, the anastomosis was observed to be broken in vivo. In these cases, the pressure was considered to be zero (BP = 0). In addition, the site of anastomosis rupture differed depending on the day (day 3 or day 7) when the in vitro bursting pressure was tested. As the healing process progresses, there is a change in collagen fibers from type III to type I. This progression starts from the point of the suture of the two bowel segments and extends laterally on both sides; furthermore, the point of change of collagen fibers is the most vulnerable point of the anastomotic area and is the one that breaks. Therefore, the earlier the tearing was after the time of suturing (day 3), the closer the tear was to the anastomosis as compared to subsequent days (day 7). Therefore, on day 3, the anastomoses ruptured at the exact point of the sutures or quite close to them, whereas on postoperative day 7, the anastomoses ruptured more circumferentially.

Finally, the excised intestinal specimens after the control of the BP and the skin–abdominal wall incisions were sent for histological observations. These pathology reports are still in progress at the time of writing.

### 2.3. Statistical Analysis

The normality of the data distribution was assessed by performing the Shapiro–Wilk test (n = 20 rats in each group). Continuous variables were reported as means and 95% confidence intervals (CIs) or as medians and interquartile ranges (IQRs) in cases of normality or non-normality, respectively. Categorical variables were presented as percentages. To compare the BP values among the groups at specific times, a one-way ANOVA was performed, and a post hoc analysis was conducted after Bonferroni correction. Based on the normality of the data, either a paired *t*-test or Wilcoxon signed-rank test was performed as a paired difference test of repeated measurements on the sample of each separate group, to compare the BP values between two different times within the same group. A Chi-square analysis was used to compare categorical variables. The statistical analysis was conducted with the Statistical Package for Social Science (SPSS), Inc. (v 25.0; Chicago, IL, USA), with the level of statistical significance set at 0.05.

## 3. Results

**Scheme 1:**
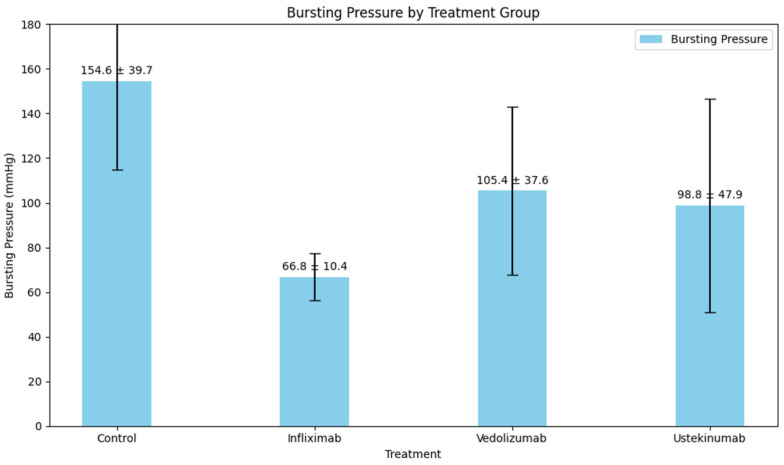
BP on postoperative day 3.

The BP values varied across the groups on POD3. The CNTRL-group developed the highest mean BP value of 154 mmHg, while the INFL-group developed the lowest one of around 66.8 mmHg. For the VDLZ-group and USTK-group, the mean BP values were 105 mmHg and 99 mmHg, respectively. The subgroup post hoc analysis revealed a statistically significant difference between the CNTRL-group and the INFL-group (154.6 mmHg ± 39.7 vs. 66.8 mmHg ± 10.4, *p* < 0.001), as well as the VDLZ-group (154.6 mmHg ± 39.7 vs. 105.4 mmHg ± 37.6, *p* < 0.001). Additionally, although marginally statistically insignificant, a difference between the CNTRL-group and the USTK-group was also noted (154.6 mmHg ± 39.7 vs. 98.8 mmHg ± 47.9, *p* = 0.051). No other differences were documented (Table 1). Thus, it is obvious that on POD3, the highest bursting pressure, and, consequently, the lowest probability of anastomosis rupture, were observed in the CNTRL-group, followed by the VDLZ-, USTK-, and INFL-groups (Scheme 1).

Moreover, significant differences were observed concerning the mortality rate. The USTK-group exhibited a significantly higher postoperative death rate on POD3 (30%) compared to the other three groups (*p* = 0.021). However, no differences were noted regarding the event of broken anastomoses among the four groups (*p* = 0.877) (Table 2).

**Scheme 2:**
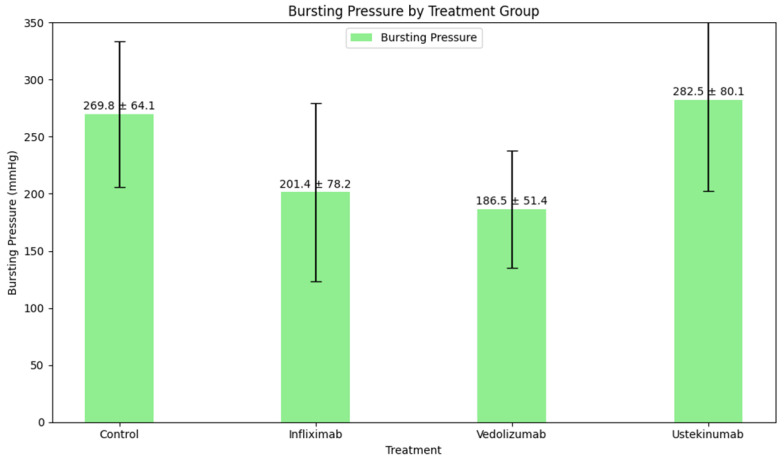
BP on postoperative day 7.

On POD7, the correlations regarding the documented BPs among the four groups changed. In particular, the previously noted differences between the CNTRL-group and both the INFL- and VDLZ-groups lost their significance, and no statistically important difference was observed between the CNTRL-group and any of the other three groups (*p* = 0.212 for the INFL-group, *p* = 0.083 for the VDLZ-group, and *p* = 1.000 for the USTK-group) (Table 3). However, in contrast to POD3, differences were found for POD7 between the INFL-group and the USTK-group (201.4 ± 78.2 mmHg vs. 282.5 ± 80.1 mmHg, *p* = 0.031) and the VDLZ-group and the USTK-group (186.5 ± 51.4 mmHg vs. 282.5 ± 80.1 mmHg, *p* = 0.031) (Table 3, Scheme 2). These changes revealed that the group that exhibited the highest BP on POD7 was the USTK-group instead of the CNTRL-group as noted for POD3. Furthermore, on POD7, no differences were found regarding the event of death or broken anastomoses among the four groups (Table 4).

**Scheme 3:**
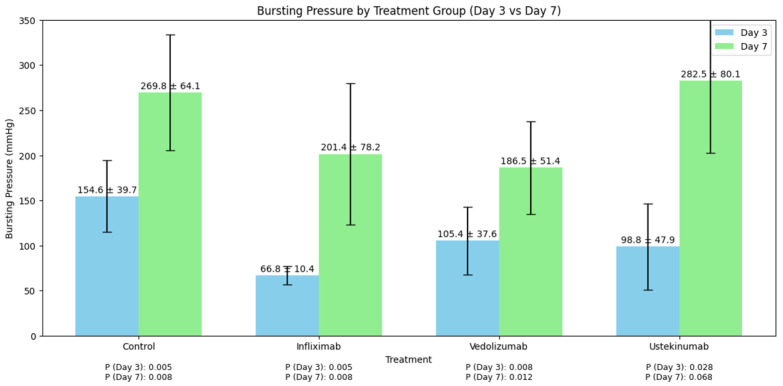
BP difference between postoperative days 3 and 7.

Finally, we compared the BP values within the same groups on POD3 and POD7 (Table 5). Two types of comparisons were made: in the first one, the anastomotic rupture was documented as a BP value of 0 mmHg; in the second one, the rats with anastomotic leakage were not included in the comparison. In all groups, the mean BP values were found to be significantly higher on POD7 compared to POD3. However, these significant differences remained only in the CNTRL-, INFL-, and VDLZ-groups when the rats exhibiting anastomotic dehiscence were excluded (Table 5). The group that showed the greatest increase in the mean BP value was the USTK-group (from 98.8 ± 47.9 mmHg to 282.5 ± 80.1 mmHg), in contrast to the VDLZ-group, in which a lower increase was observed (from 105.4 ± 37.6 to 186.5 ± 51.4) (Scheme 3).

## 4. Discussion

The strength of anastomoses is assessed on the basis of their bursting pressure (BP), with higher BP values indicating stronger anastomoses during the first week after surgery. The bursting pressure is defined as the internal pressure at which an anastomosis fails, making it a critical measure of the mechanical strength of these surgical junctions. Chlumsky [20] first employed this method in 1899. From 1964 onward, more consistent data emerged, primarily focused on comparing different suturing techniques. During the first 3 to 4 days, known as the lag period, the bursting pressures remain low. Following this period, the BP increases rapidly to levels significantly higher than those observed in “uninjured” intestines [21]. This pattern has also been observed in the large [20,21] and small intestines [22,23] of rats. In essence, intestinal BP refers to the maximum pressure the walls of the intestine can withstand before mechanical failure occurs, potentially leading to perforation or rupture. As a result, BP is considered a key factor in evaluating the effectiveness of intestinal sutures [24].

Plenty of studies have assessed the complications associated with the perioperative administration of infliximab, as compared to patients receiving no agents at the time of surgery or administered other biological factors. In general, in comparisons with groups of patients who did not receive any agents, the results in the majority were not statistically significant [25]. A statistically significant increase in inflammatory complications [26] was observed relative to overall complications, mortality, and reinterventions [27]. Despite these results, El Hussuna et al. [28] concluded that studies with limited bias showed an increase in anastomotic complications in patients treated with anti-TNFs (RR 1.63, 95% CI 1.03–2.60). On the other hand, further studies showed similar results concerning postoperative outcomes, although the difference was statistically insignificant [29]. Additionally, a case-matched study compared the effect of infliximab with a control group and concluded that there was no statistically significant difference in postoperative outcomes between the two groups. This result is in contrast to the outcomes of our experimental protocol, where a difference in the anastomosis bursting pressure was observed between the infliximab and control groups on day 3 after the operation [9]. In an experimental study similar to ours, a difference in complications related to anastomoses was observed between a group of patients receiving perioperative anti-TNF induction therapy and a control group; this result, like ours, is in contrast to the literature, in which the differences between these values are not significant [9].

As previously described, the vedolizumab and control groups showed differences in terms of bursting pressure, with the control group showing higher values on both day 3 and day 7. However, this effect was significant only on postoperative day 3. In a 2018 review, no significant difference was observed between these two comparative groups in terms of either overall or inflammatory complications [10].

No acceptable difference was described between anti-TNF agents (infliximab) and vedolizumab in patients [10], either. In comparisons of complications at days 30 and 90 post-intervention, a trend of decrease in the rate of anastomotic leakage in the vedolizumab group was observed, as well as a decrease in inflammatory complications, which also did not turn out to be statistically significant (*p* = 1) [30]. The corresponding results in our study showed variation in the value of the bursting pressure between the two groups, both on day 3 and on day 7, but without a significant difference. Additionally, the VDLZ-group predominated on day 3, a fact that was reversed on postoperative day 7. In general, for patients with IBDs, no differences are observed in terms of their overall postoperative complications with reference to the intake of infliximab or vedolizumab. However, the rates may differ in patients with ulcerative colitis: in such patients, VDLZ has shown reduced complication rates compared to patients with Crohn’s disease, in whom any difference is insignificant [17].

A 2021 meta-analysis compared the impact of the perioperative administration of ustekinumab with that of anti-TNF agents, concluding that the results are still ambiguous [12]. In most studies, no differences were observed for USTK in comparison with both anti-TNF agents and VDLZ [12,17]. The meta-analysis compared the impact of perioperative USTK (189 patients) and an anti-TNF factor (481 patients), and concluded that there were no differences in septic complication rates between the two groups (7.2% and 11.9%, respectively; *p* = 0.4) [12]. The results of our protocol, as presented in Table 1 and Table 2, seem to show a difference in the bursting pressure between group B of infliximab-induced rats and group D, to whom we administered ustekinumab. The mean values for USTK remained higher than those for infliximab, but they were statistically significant only on day 7.

An observational study that compared patients administered anti-TNF and USTK prior to surgery concluded that despite the fact that the ustekinumab group (20 patients) showed particularly high rates of stoma (70% vs. 12.5%, *p* < 0.001) and an increased likelihood of needing further immunosuppression therapy, the differences in terms of surgical complications were not important. More precisely, the rates of anastomotic leak were 0 for the USTK group and 7.5% for the anti-TNF group, and their difference was not significant (*p* = 0.54); similar results were found for the rates of postoperative ileus and wound infections [13].

Finally, in the comparison of preoperative vedolizumab administration with ustekinumab treatment, as presented in Table 1 and Table 2, the bursting pressure in the ustekinumab group on day 7 after surgery showed a huge increase compared with both the corresponding value on day 3 and the corresponding values for the other groups. In a matched-case analysis from 2018 with 103 patients, it was shown that vedolizumab was associated with increased rates of complications and postoperative ileus (*p* = 0.009 and *p* = 0.015) compared to ustekinumab. However, after logistic regression, the difference was found to be statistically insignificant [31].

In our experiments, we observed that rats receiving VDZ as the biological agent showed intra-abdominal adhesions, both between intestinal loops and with the abdominal wall, independently of death or anastomosis leak. This fact resembles the conclusions of several other studies reporting that vedolizumab increases inflammatory complications and SSIs [32] or is possibly responsible for increased overall morbidity in patients as compared to a control or infliximab group. Furthermore, we observed that 14 out of the 20 rats that received UST presented wound dehiscence. This resembles the result of a study in which USTK was found to increase the likelihood of intraperitoneal sepsis compared to a control group [33]. However, this result is in contrast with that of a multicenter study in which the incidence of intra-abdominal inflammation after the administration of ustekinumab did not differ from that in the other groups [34,35].

In conclusion, concerning the strengths of this study, all the experiments were completed by one researcher. Furthermore, as already mentioned above, this is the first experimental study comparing three different biological agents according to their direct impact on intestinal anastomoses. On the other hand, as a limitation of this protocol, the experimental procedure was not blinded, as the researcher knew in advance which agent was administered in which rat. Furthermore, although the surgical intervention was performed by the same person, the surgical technique still depended on human factors, since the anastomoses were performed by hand. This limitation could not be avoided, as due to the size of the animals it was impossible to use any automatic tool to perform the anastomoses. Moreover, while our study focused on evaluating the therapeutic effects of each antibody on its specific target, we acknowledge that humanized antibodies can elicit immune responses in non-human species, potentially influencing the observed outcomes [36]. The study design, however, was based on pre-existing experimental models in which the control group did not receive any treatment. Ultimately, if the aforementioned theory was entirely accurate, the control group would demonstrate the most favorable outcomes across all comparisons and time intervals; however, this was not reflected in the results of our study. Nevertheless, we propose incorporating an isotype control antibody in future experiments. Furthermore, we acknowledge that our study lacks histological data, collagen fiber assessments, and molecularly measurable markers reflecting the healing process (e.g., TGF, VEGF, interleukins); these are being processed at the time of writing. Additionally, it is important to note as a limitation of this study that the animals did not have established colitis, unlike the patients theoretically receiving the corresponding treatments in this context. However, the comparisons between the groups were conducted under similar conditions (normal intestines for all the rat groups), and, as such, the procedure for comparing the results was not compromised. A follow-up study is planned to include animals with induced colitis, and the results of that study will be compared with those of the present one.

## 5. Conclusions

The results of this experimental study highlight the varying detrimental effects of different biological agents on the resistance of intestinal anastomoses. While this topic has been explored in numerous studies, no definitive conclusions have been reached. The international literature acknowledges differences between biological agents, although these differences are often not statistically significant in a majority of the studies.

The growing recognition within the international research community of the need for further studies on the postoperative complications associated with biological agents is critical. As biological therapies continue to advance, understanding their implications in the perioperative setting is essential for optimizing patient outcomes. Future research should prioritize comprehensive studies that assess both short-term and long-term outcomes of biological interventions across diverse surgical populations.

## Figures and Tables

**Table 1:** Bursting pressure values on postoperative day 3.

Type of Comparison		*p*-Value
One-way ANOVA		<0.001
Subgroup Analysis	Bursting Pressure (mmHg) [mean ± SD]	
Control vs. infliximab	154.6 ± 39.7 vs. 66.8 ± 10.4	<0.001
Control vs. vedolizumab	154.6 ± 39.7 vs. 105.4 ± 37.6	<0.001
Control vs. ustekinumab	154.6 ± 39.7 vs. 98.8 ± 47.9	0.051
Infliximab vs. vedolizumab	66.8 ± 10.4 vs. 105.4 ± 37.6	1.000
Infliximab vs. ustekinumab	66.8 ± 10.4 vs. 98.8 ± 47.9	0.207
Vedolizumab vs. ustekinumab	105.4 ± 37.6 vs. 98.8 ± 47.9	0.365

**Table 2:** Mortality and anastomotic leak rates on postoperative day 3.

Event	Group	Observed	Not Observed	*p*-Value
Mortality	Control	0	10	0.021
	Infliximab	0	10	
	Vedolizumab	0	10	
	Ustekinumab	3	7	
Anastomotic Leak	Control	1	9	0.877
	Infliximab	1	9	
	Vedolizumab	1	9	
	Ustekinumab	2	8	

**Table 3:** Bursting pressure values on postoperative day 7.

Type of Comparison		*p*-Value
One-way ANOVA		0.008
Subgroup Analysis	Bursting Pressure (mmHg) [mean ± SD]	
Control vs. infliximab	269.8 ± 64.1 vs. 201.4 ± 78.2	0.212
Control vs. vedolizumab	269.8 ± 64.1 vs. 186.5 ± 51.4	0.083
Control vs. ustekinumab	269.8 ± 64.1 vs. 282.5 ± 80.1	1.000
Infliximab vs. vedolizumab	201.4 ± 78.2 vs. 186.5 ± 51.4	1.000
Infliximab vs. ustekinumab	201.4 ± 78.2 vs. 282.5 ± 80.1	0.031
Vedolizumab vs. ustekinumab	186.5 ± 51.4 vs. 282.5 ± 80.1	0.031

**Table 4:** Mortality and anastomotic leak rates on postoperative day 7.

Event	Group	Observed	Not Observed	*p*-Value
Mortality	Control	0	10	0.380
	Infliximab	0	10	
	Vedolizumab	1	9	
	Ustekinumab	0	10	
Anastomotic Leak	Control	0	10	N/A
	Infliximab	0	10	
	Vedolizumab	0	10	
	Ustekinumab	0	10	

N/A: cannot be calculated.

**Table 5:** Comparisons within the same groups between postoperative day 3 and postoperative day 7.

Group	Dehiscence Pressure (mmHg) [mean ± SD]		*p*-Value *	*p*-Value ^#^
	Day 3	Day 7		
Control	154.6 ± 39.7	269.8 ± 64.1	0.005	0.008
Infliximab	66.8 ± 10.4	201.4 ± 78.2	0.005	0.008
Vedolizumab	105.4 ± 37.6	186.5 ± 51.4	0.008	0.012
Ustekinumab	98.8 ± 47.9	282.5 ± 80.1	0.028	0.068

* Broken anastomoses were measured as a pressure of 0. # Broken anastomoses were not included.

## Data Availability

The raw data supporting the conclusions of this article will be made available by the authors on request.

## Abbreviations

The following abbreviations are used in this manuscript:

POD	Postoperative day
BP	Bursting pressure
IBDs	Inflammatory bowel diseases
INFL	Infliximab
VDLZ	Vedolizumab
USTK	Ustekinumab
